# Sleep, Chronotype and Physiological Acclimatisation During a 21-Day Altitude Training Camp in World-Class Swimmers

**DOI:** 10.3390/sports14100449

**Published:** 2026-10-11

**Authors:** José Francisco Alonso Ramos, José Antonio Del Castillo, Alejandro López-Hernández, José María González-Ravé

**Affiliations:** 1 Sports Training Laboratory, Faculty of Sports Sciences, University of Castilla La Mancha, Carlos III Avenue, 5, 45071 Toledo, Spain; alejandro.lopez@uclm.es (A.L.-H.); josemaria.gonzalez@uclm.es (J.M.G.-R.); 2 Club Natació Sabadell, 08203 Sabadell, Spain; kasti71@gmail.com

**Keywords:** hypoxia, chronotype, sleep, altitude training, acclimatisation, elite swimmers

## Abstract

Altitude training is widely used in elite swimming, although its effects on sleep and acclimatisation remain insufficiently understood. The aim of this study was to describe changes in sleep difficulty, physiological acclimatisation, athlete-reported outcomes, and haematological markers during a 21-day altitude training camp (2320 m), and to explore the association between chronotype and sleep difficulty. Seven world-class swimmers (4 men, 3 women) were monitored before, during, and after altitude exposure. Sleep difficulty was assessed using the Athlete Sleep Screening Questionnaire, while daily monitoring included resting oxygen saturation, resting heart rate, acute mountain sickness symptoms, fatigue, recovery, hydration status, and training load. Chronotype was determined using the Morningness-Eveningness Questionnaire. Sleep difficulty was descriptively higher during the first week at altitude (6.14 ± 1.07 vs. 4.14 ± 1.21 at baseline) and returned towards baseline by week 3 (4.29 ± 1.50), although pairwise comparisons did not remain significant after multiplicity correction. Strong inverse associations between chronotype and sleep difficulty were observed at baseline (rho = −0.87, *p* = 0.014) and week 1 (rho = −0.85, *p* = 0.019), but neither met the Bonferroni-adjusted significance threshold (α = 0.010). SpO_2_ increased across the camp, with week 3 significantly higher than weeks 1 and 2 after Bonferroni correction, whereas RHR was significantly lower in week 3 than in week 1, but not week 2, after correction. Athlete-reported outcomes remained stable, acute mountain sickness symptoms were minimal, and haematological and iron-status markers showed no significant pre-to-post changes. Sleep difficulty showed a transient descriptive increase during the initial phase of the altitude camp, while SpO_2_ and RHR changes were consistent with physiological acclimatisation. Chronotype-related differences in sleep difficulty appeared to be present before altitude exposure rather than emerging specifically during the camp. Given the small sample size, these findings should be considered exploratory and hypothesis-generating.

## 1. Introduction

Altitude training is a standard component of preparation for elite swimmers. Training camps at moderate-altitude centres are routinely incorporated into world-class performance programmes [1,2]. The physiological rationale is well established, as hypoxic exposure induces hematological and non-haematological adaptations that may contribute to improved sea-level performance [3,4,5].

Despite these potential benefits, altitude exposure also imposes additional physiological stress that may compromise recovery processes. Among the most frequently reported consequences is sleep disturbance. Increased ventilatory drive under hypoxic conditions can promote nocturnal micro-arousals and reduce sleep continuity, particularly during the first days of exposure [6,7]. In addition, reductions in nocturnal oxygen saturation have been associated with greater sleep fragmentation [7], while symptoms of acute mountain sickness, typically most pronounced within the first 24–72 h of exposure, may further aggravate these disturbances [8,9].

Sleep is widely recognised as a fundamental component of athletic recovery and performance. Inadequate sleep has been associated with impaired physical performance, mood disturbances and reduced recovery capacity between training sessions [10,11]. Consequently, the systematic monitoring of sleep has become an essential aspect of athlete management, particularly during periods characterised by increased training loads or exposure to environmental stressors [10,12].

Among these stressors, altitude exposure represents a unique challenge due to its potential to disrupt sleep and recovery processes. Although altitude training is frequently implemented in elite sport, its specific impact on sleep in highly trained athletes with previous altitude experience remains insufficiently understood. Indeed, much of the available evidence originates from laboratory-based studies or investigations conducted in non-athletic populations, limiting its direct applicability to elite sporting contexts [7,13].

Alongside sleep monitoring, the assessment of athlete-reported wellbeing, fatigue, and recovery has become increasingly important during training camps and periods of intensified training. Athlete-reported outcome measures have demonstrated sensitivity to fluctuations in training status and overall wellbeing, providing practitioners with valuable information to detect maladaptive responses and guide training decisions [10,12]. Therefore, the combined assessment of sleep, physiological acclimatisation, and perceptual responses may offer a more comprehensive understanding of athletes’ adaptation to altitude exposure.

Chronotype, defined as an individual’s preferred timing of sleep and daily activities, has emerged as a relevant factor in sleep and performance research [14,15]. Nevertheless, its potential influence on adaptation to altitude remains largely unexplored. Specifically, it is unknown whether athletes with different chronotypes exhibit differential susceptibility to hypoxia-related sleep disturbances or distinct patterns of physiological and perceptual adaptation during altitude exposure.

Longitudinal and multidimensional monitoring studies conducted in real-world altitude training camps involving elite athletes remain scarce, largely due to the limited accessibility of high-performance populations under ecologically valid conditions [16]. This scarcity highlights the value of detailed observational data from elite cohorts, which can provide important preliminary evidence and generate hypotheses for future, adequately powered investigations.

Therefore, the aim of this study was to describe the evolution of sleep difficulty, physiological markers of acclimatisation (peripheral oxygen saturation (SpO_2_) and resting heart rate (RHR), athlete-reported indicators of recovery and fatigue, and haematological parameters during a 21-day altitude training camp (2320 m) in seven international-level swimmers. Based on previous altitude and chronobiology literature, it was hypothesized that: (1) hypoxia exposure would induce a transient disruption in sleep quality and acute physiological responses, peaking during the first week and progressively recovering alongside acclimatisation; and (2) baseline chronotype would be associated with individual differences in sleep difficulty, with evening-type athletes exhibiting higher sleep difficulty, particularly during the initial phase of altitude exposure.

## 2. Materials and Methods

### 2.1. Participants

Seven world-class swimmers (4 men, 3 women; age: 21.00 ± 2.80 years, height: 182.46 ± 9.60 cm, body mass: 75.12 ± 12.43 kg; all ≥18 years) from Club Natación Sabadell volunteered to participate in the study. All swimmers were integrated into a national-level high-performance program and had previous exposure to altitude training as part of their annual periodization. Competitive level, evaluated via official World Aquatics point tables, was 849 ± 50.70 AQUA points, corresponding to Tier 4–5 criteria according to McKay et al. [17]. Inclusion criteria were elite status (Tier 4–5), age ≥ 18 years, and completion of at least one altitude camp within the two preceding competitive seasons. Exclusion criteria included any medical contraindication for altitude exposure or sport-related issues requiring premature withdrawal from the camp. Prior to baseline testing, athletes received a full explanation of study procedures and provided written informed consent. Given the restricted pool of athletes meeting these criteria, the sample size reflects the inherent constraints of accessing elite swimmers under ecologically valid, high-performance conditions, consistent with prior work in this population [16].

### 2.2. Study Design

A prospective case-series study with longitudinal repeated measures was used to examine the temporal evolution of sleep, recovery, training load, hypoxia related symptoms, and selected physiological markers. The study was conducted in real-world, high-performance conditions without altering athletes’ training programs, recovery strategies, or medical supervision practices. Monitoring occurred across three phases: (1) a baseline period during the week preceding altitude exposure; (2) a 21-day altitude training camp at the Sierra Nevada High Performance Center (2320 m); and (3) a 7 day post-altitude phase following return to sea level. Daily perceptual and physiological monitoring was confined to the altitude phase, whereas sleep quality via the Athlete Sleep Screening Questionnaire (ASSQ) was assessed at all three stages to characterize global patterns before, during, and after altitude exposure (Figure 1).

### 2.3. Procedures

Before data collection began, athletes were briefed individually on measurement protocols and questionnaire requirements to ensure consistency across the monitoring period. At baseline, participants completed the Morningness-Eveningness Questionnaire (MEQ) to determine chronotype [18]. The baseline ASSQ assessment was completed upon arrival at altitude, before the first night of altitude exposure, and retrospectively referred to sleep during the preceding seven days at sea level. Pre-altitude blood biomarkers provided by the medical team served as reference indicators for individual physiological status.

During the altitude camp, measurements were performed twice daily. Morning monitoring occurred immediately after waking and before breakfast, comprising objective physiological markers (resting SpO_2_ and RHR) and daily self-reported metrics (hypoxia symptoms via the Lake Louise Score, fatigue via the TSF-10, hydration status through urine color, and perceived recovery). Evening monitoring occurred following the final training session and captured training load parameters (session volume, session type, and rating of perceived exertion (RPE)). Global sleep difficulty was tracked weekly using the ASSQ during the week preceding altitude exposure (baseline), weekly throughout the altitude phase, with a final assessment completed during the 7-day post-altitude phase to evaluate sleep patterns upon returning to sea level.

### 2.4. Measures

Resting heart rate was obtained using a Polar H10 chest strap each morning in a standardized supine position. The Polar H10 has been validated against electrocardiogram (ECG) [19]. Resting oxygen saturation (SpO_2_) was assessed using a Contec CMS50D fingertip pulse oximeter following the same timing and posture as RHR. This device has been clinically validated against hospital-grade reference monitors [20]. Sleep difficulty was evaluated using the Athlete Sleep Screening Questionnaire [21]. The ASSQ was selected because it is a brief, athlete-specific screening tool validated to capture perceived sleep difficulty in a training context, rather than to provide objective measures of sleep architecture such as sleep stages or continuity. Hypoxia related symptoms were assessed via the revised Lake Louise Score [22]. Daily perceived fatigue was measured using the Total Score of Fatigue (TSF-10) [23]. Perceived recovery was assessed each morning using a 0–10 scale based on the Total Quality Recovery Scale [24]. Chronotype was classified using the Morningness-Eveningness Questionnaire MEQ [18]. Hydration status was assessed daily via urine color. Training load was characterized using weekly in-water training volume. Internal training load was quantified using the session-RPE method [25,26]. For each training session, sRPE-min was calculated by multiplying the athlete’s session RPE by the session duration in minutes and was expressed in arbitrary units (AU). For each athlete, session-level sRPE-min values were averaged across all training sessions completed within each week to obtain an individual weekly mean sRPE-min. Group values were subsequently calculated as the mean ± SD of these individual weekly means. All data were collected using Microsoft Forms and securely stored on institutional OneDrive.

Haematological data were obtained from the athletes’ routine medical monitoring programme conducted by the club’s medical staff as part of their standard physiological follow-up. Venous blood samples (4 mL) were collected from an antecubital vein in the morning, 3 days before the altitude training camp and on the first day after returning to sea level. The laboratory reports provided to the research team included haemoglobin concentration, haematocrit, erythrocyte count, serum iron, transferrin, transferrin saturation, and ferritin. All biomarker values reported in the present study were obtained directly from the clinical laboratory reports; no haematological or iron-status variables were derived or calculated by the research team. Detailed information regarding the analytical platform and assay procedures for all biomarkers was not available to the research team. These data were used to contextualise the physiological responses observed during the study.

### 2.5. Ethical Approval

The protocol was approved by the Social Research Ethics Committee of the University of Castilla-La Mancha (CEIS-2026-186014). All procedures complied with the Declaration of Helsinki and applicable data protection regulations. Data were pseudonymized using numerical identifiers stored separately from analytical files.

### 2.6. Statistical Analysis

Data were analyzed using a descriptive, exploratory, intra individual approach consistent with the small elite sample (*n* = 7). Because resting SpO_2_ and resting heart rate were recorded daily during the altitude phase, whereas sleep difficulty was assessed weekly, daily values for these two variables were averaged within each week to obtain a single mean value per athlete per week, matching the weekly time points used for the remaining variables. Means and standard deviations were calculated for all variables. Normality was assessed with the Shapiro–Wilk test. Temporal changes were evaluated using Friedman tests. When a significant overall effect was detected, pairwise comparisons were performed using exact two-tailed Wilcoxon signed-rank tests. Pairs with zero difference were excluded from the test, as is standard in the Wilcoxon procedure. Following the significant Friedman test for SDS, four planned pairwise comparisons were performed using two-tailed Wilcoxon signed-rank tests, comparing week 1 with PRE, week 2, week 3, and POST. To account for these four comparisons, statistical significance was assessed using a Bonferroni-corrected threshold of α = 0.0125 (0.05/4). Associations between chronotype and sleep difficulty were examined using Spearman’s rank correlations. Given the small sample size (*n* = 7), two-sided exact permutation *p*-values were obtained by enumerating all possible permutations of the seven paired observations. Confidence intervals for Spearman’s rho were calculated using Fisher’s z transformation and back-transformed to the correlation scale. To account for the five chronotype–sleep difficulty correlations, statistical significance was evaluated using a Bonferroni-adjusted threshold of α = 0.010 (0.05/5). Effect sizes for Wilcoxon signed-rank tests were calculated using Rosenthal’s r (r = |Z|/√N), where Z is the standardized Wilcoxon test statistic and N is the number of non-zero paired differences, and were interpreted as small (0.10), moderate (0.30), or large (≥0.50) according to conventional criteria [27]. Analyses were conducted using IBM SPSS Statistics (Version 26, IBM Corp., Armonk, NY, USA). Given the small sample size, emphasis was placed on the magnitude and direction of effects rather than statistical significance alone.

## 3. Results

### 3.1. Evolution of Sleep Difficulty

Sleep difficulty peaked during the first week at altitude (6.14 ± 1.07) compared with the pre-altitude baseline (4.14 ± 1.21), before returning to values close to baseline during weeks 2 (4.57 ± 1.72) and 3 (4.29 ± 1.50) (Figure 2). A significant overall effect across time points was observed (χ^2^(4) = 10.82, *p* = 0.03). Four planned pairwise comparisons were subsequently performed between week 1 and each of the other assessment points. None reached the Bonferroni-corrected significance threshold of α = 0.0125: W1 vs. PRE (W = 0.0, *p* = 0.0156, r = 0.91), W1 vs. W2 (W = 2.5, *p* = 0.0781, r = 0.75), W1 vs. W3 (W = 0.0, *p* = 0.0313, r = 0.91), and W1 vs. POST (W = 5.0, *p* = 0.1563, r = 0.60). These results indicate a descriptively higher sleep-difficulty score during the first week at altitude, with large effect sizes, although none of the individual pairwise comparisons met the corrected significance threshold.

### 3.2. Chronotype and Sleep Difficulty

Strong inverse correlations between chronotype and sleep difficulty were observed at pre-altitude baseline (rho = −0.87, *p* = 0.014) and during week 1 at altitude (rho = −0.85, *p* = 0.019). However, neither association met the Bonferroni-adjusted significance threshold (α = 0.010). Correlations at subsequent assessment points were weaker and did not reach statistical significance (Table 1; Supplementary Figure S1). Given the small sample size, these associations are interpreted as exploratory estimates based primarily on their magnitude and direction.

### 3.3. Physiological Acclimatisation: SpO_2_ and Resting Heart Rate

Resting oxygen saturation and resting heart rate changed significantly across the three weeks at altitude, with a significant main effect of time for both variables (Friedman test, *p* ≤ 0.01; Table 2). Pairwise comparisons showed that SpO_2_ was significantly higher in week 3 than in week 1 (Wilcoxon W = 0.0, Bonferroni-adjusted *p* = 0.047, r = 0.89) and week 2 (W = 0.0, adjusted *p* = 0.047, r = 0.89), whereas the week 1–week 2 comparison was not significant after correction (W = 4.0, adjusted *p* = 0.656, r = 0.56; one athlete showed no change between weeks 1 and 2, so this comparison was based on *n* = 6 non-zero differences). For RHR, week 3 was significantly lower than week 1 (W = 0.0, adjusted *p* = 0.047, r = 0.89), whereas the week 1–week 2 (W = 1.0, adjusted *p* = 0.094, r = 0.83) and week 2–week 3 comparisons (W = 7.0, adjusted *p* = 0.891, r = 0.45) did not remain statistically significant after correction.

Individual trajectories for both variables further illustrate this uniform acclimatisation pattern across athletes (Figure 3).

### 3.4. Athlete-Reported Outcomes: Symptoms, Fatigue, Hydration, and Recovery

Athlete-reported outcome measures remained stable across the 21-day altitude camp, with no statistically significant weekly changes observed for any variable (Friedman test, all *p* > 0.05). Symptoms of acute mountain sickness evaluated via the Lake Louise Score remained minimal throughout the stay. Similarly, perceived fatigue (TSF-10 total score), hydration status, and perceived recovery showed no meaningful fluctuations across the three weeks, indicating that athletes well tolerated the overall altitude and training load stress (Table 3).

### 3.5. Training Load Parameters

Weekly training load parameters are summarized in Table 4. In-water training volume remained elevated during weeks 1 and 2, followed by a marked reduction in week 3 (Table 4). Perceived exertion decreased progressively across the camp, from 7.11 ± 0.41 in week 1 to 5.67 ± 0.36 in week 2 and 5.29 ± 0.28 in week 3. In contrast, sRPE-min decreased markedly from week 1 (1441.12 ± 77.85 AU) to its lowest value in week 2 (878.68 ± 57.76 AU), followed by a slight increase in week 3 (901.69 ± 53.52 AU). Exploratory correlation analyses between weekly training load metrics and physiological or perceptual response variables revealed no consistent or systematic patterns across time points (Supplementary Figure S2).

### 3.6. Blood Biomarkers

Pre- to post-altitude changes in haematological and iron-status parameters are presented in Table 5. No statistically significant changes were detected in haemoglobin concentration, haematocrit, or ferritin levels following the 21-day camp (all *p* > 0.30; Table 5). Overall, blood markers remained stable within baseline physiological ranges across the cohort.

## 4. Discussion

This study provides one of the few longitudinal, multidimensional descriptions of sleep, physiological acclimatisation, and perceptual responses during a real-world altitude camp in world-class swimmers. Three findings stand out: (1) sleep difficulty was descriptively higher during the first week at altitude, with large effect sizes despite the loss of significance after correction for multiple comparisons; (2) strong negative correlations between morningness and sleep difficulty were observed before altitude exposure and during week 1, although neither remained statistically significant under the Bonferroni-adjusted threshold, suggesting descriptively that this pattern may reflect a pre-existing rather than altitude-induced vulnerability; and (3) SpO_2_ and resting heart rate showed changes consistent with physiological acclimatisation across the three weeks, although not all pairwise comparisons remained statistically significant after correction, while athlete-reported outcome measures and haematological markers remained stable.

The pattern of sleep disruption observed here is consistent with previous reports linking the early days of altitude exposure to increased nocturnal arousals and reduced sleep continuity [7]. The fact that the effect was largest in week 1 and attenuated thereafter fits the classical description of an initial ventilatory-acclimatisation window, during which hypoxic drive is highest before partial habituation occurs [28]. Although statistical power was limited by the elite cohort size (*n* = 7), the consistency of direction and the large effect sizes (r = 0.75–0.91) are descriptively consistent with an early increase in sleep difficulty upon arrival at altitude.

The chronotype findings address a novel question regarding individual adaptation in high-performance altitude environments. Strong inverse correlations between morningness and sleep difficulty were observed at pre-altitude baseline (rho = −0.87, *p* = 0.014) and during week 1 at altitude (rho = −0.85, *p* = 0.019). Neither association met the Bonferroni-adjusted significance threshold (α = 0.010). Considered descriptively, this pattern suggests that the association between morningness and sleep difficulty was already evident before altitude exposure and remained similar in magnitude during the first week at altitude. Correlations were weaker at subsequent assessment points; however, given the small sample size and the absence of formal comparisons between correlation coefficients across time, these later estimates should not be interpreted as evidence that the chronotype–sleep association disappeared. A possible interpretation is that eveningness-related sleep difficulty may reflect a pre-existing individual characteristic rather than an effect specifically induced or amplified by altitude. One likely reason is the camp’s schedule: athletes were woken by staff at a fixed time each morning, while bedtime was not standardised, so evening-type athletes going to bed later by natural preference would have had less sleep before the same fixed wake-up, a kind of social jet lag that could explain their extra sleep difficulty on its own, independent of altitude. However, habitual sleep and wake times outside the training camp were not recorded; therefore, this interpretation remains speculative and requires further investigation. If the association between chronotype and sleep difficulty is confirmed in larger cohorts, pre-camp chronotype screening using instruments such as the MEQ may help identify athletes who could benefit from individualised sleep-management strategies. These strategies could include targeted sleep-hygiene interventions or adjustments to training schedules, rather than implementing uniform recommendations across the entire team.

The trajectory of SpO_2_ and RHR mirrors the acclimatisation timeline reported in previous altitude-training [3] and its consistency across athletes, despite the marked individual variability typically reported in hypoxic responses, reinforces the value of within-athlete repeated monitoring over single-timepoint assessment. The absence of significant change in Lake Louise scores, TSF-10 fatigue, hydration status, or perceived recovery suggests that, at a group level, the camp was well tolerated beyond the sleep domain. This finding is consistent with the athletes’ prior altitude experience, as all participants had attended at least one previous altitude training camp, and repeated hypoxic exposures have been shown to attenuate acute mountain sickness symptoms in subsequent altitude sojourns [8]. It is plausible that this prior exposure also contributed to milder sleep disruption, although this specific link is less well established in the literature than its effect on acute mountain sickness symptoms. However, in the absence of a sea-level control group, these physiological changes should be interpreted as consistent with expected altitude acclimatisation rather than as direct evidence of the training camp’s effectiveness, since a similar temporal pattern could not be fully distinguished from concurrent training-cycle effects.

The absence of significant change in haematological and iron-status markers over 21 days should be interpreted with caution rather than as evidence of non-adaptation. Gore et al. [29] estimated an increase in haemoglobin mass of approximately 1.1% per 100 h of adequate altitude exposure and concluded that classic altitude camps as short as two weeks are likely to increase haemoglobin mass. Importantly, haemoglobin mass was not measured in the present study; the available clinical data comprised concentration-based haematological markers and iron-status indices. Therefore, unchanged haemoglobin concentration and haematocrit should not be interpreted as evidence that haemoglobin mass or the underlying erythropoietic response did not change. Similar altitude camps in elite swimmers have reported similarly unchanged haemoglobin and haematocrit alongside detectable underlying erythropoietic activity captured only through more sensitive indices such as reticulocyte counts, ferritin dynamics, or haemoglobin mass [30,31]. In our data, the absence of detectable changes may therefore reflect measurement timing and method and should not be interpreted as evidence of absent physiological adaptation.

One thing worth discussing separately is how training load evolved. Training-load metrics did not follow identical trajectories across the camp. In-water training volume remained high during weeks 1 and 2 before decreasing markedly in week 3, while RPE decreased progressively across the three weeks. In contrast, sRPE-min decreased markedly from week 1 to its lowest value in week 2, followed by a slight increase in week 3. Because training stress and hypoxic acclimatisation were not manipulated independently in this real-world case-series design, the concurrent changes in training load cannot be fully separated from altitude acclimatisation as potential contributors to the physiological changes observed. Coaches typically reduce training volume toward the end of an altitude camp as part of competition preparation, consistent with established tapering practices in swimming [32]. Consequently, the more favourable SpO_2_ and RHR values observed in week 3 may have been influenced not only by altitude adaptation but also by the concurrent reduction in training load. Future studies should control for training load, either through a fixed training programme or statistical adjustment, to better distinguish the effects of training load from those of altitude exposure. Similarly, the exploratory correlations between training load metrics and sleep or physiological variables (Supplementary Figure S2) should be read as hypothesis-generating observations rather than confirmed relationships, given the large number of comparisons performed relative to the sample size.

Comparisons with the limited literature on sleep during real-world altitude camps in elite swimmers should be made cautiously. In a 14-day altitude camp at 1500 m involving Olympic-level open-water swimmers, Vitale et al. [33] found no significant changes in objective or subjective sleep-quality measures across the camp. They did, however, report earlier sleep onset and offset during the first two days of altitude exposure compared with baseline. Differences in altitude, sleep assessment methods, and study design limit direct comparison with the present findings. Similarly, Belluco et al. [34], in a case report of a Paralympic record-breaking athlete and his guides, documented that self-reported sleep quality and acute mountain sickness symptoms were most pronounced early in altitude exposure and improved thereafter, reinforcing the generalisability of an early-adaptation sleep-disruption window across different elite populations and altitude modalities. Taken together, these observations support the possibility that the initial days of altitude exposure may represent a period of increased sleep vulnerability across different elite-athlete populations and altitude-training contexts.

This study has several limitations that should be considered when interpreting the findings. The sample size (only seven elite swimmers), while consistent with the scarcity of ecologically valid elite-athlete data in this field [16], limits statistical power and the stability of correlation estimates. We included both men and women, but a sample this small (4 men, 3 women) did not allow us to properly test for sex differences, so men and women were analysed together, as a practical necessity, not because we assume they respond identically to altitude. To provide some information on possible sex-related differences, descriptive data are presented separately in the Supplementary Material (Tables S1–S6), and individual trajectories are shown by sex in Figure 2. The small sample also increases the risk of both Type I and Type II errors across the multiple correlation and comparison analyses performed. While Bonferroni correction was applied to reduce the likelihood of false-positive findings, this same correction, combined with the limited statistical power of *n* = 7, increases the risk of failing to detect associations that may nonetheless be real (Type II error). Sleep and symptom data relied on self-report instruments, which, although validated, are subject to reporting bias. Furthermore, we did not collect objective or quantitative sleep characteristics such as total sleep time, sleep efficiency, sleep onset latency, or wake after sleep onset, which limits the ability to characterise the specific nature of the sleep disturbances observed. In addition, the pre-altitude (PRE) ASSQ assessment was completed on arrival at altitude and relied on athletes’ memory recall of the preceding week at sea level, rather than daily recording before departure; sleep during that baseline week may already have been affected by anticipation of the upcoming camp. Subsequent ASSQ assessments (weeks 1–3 and post-altitude) were completed prospectively at the end of each week.

Hydration status was assessed using a subjective urine colour chart, which may have limited the sensitivity of this measure. Finally, the absence of a sea-level control group monitored over an equivalent period means that some of the observed temporal changes cannot be fully disentangled from training-cycle or seasonal effects unrelated to altitude itself.

Future studies should examine the relationship between chronotype and altitude-related sleep disturbances in larger cohorts of elite athletes and across different sporting disciplines. Using accompanying, non-competing staff as a sea-level or lower-altitude control group could also help separate altitude-specific effects from training-cycle or seasonal ones. The inclusion of objective sleep measures, such as actigraphy or polysomnography, would provide a more comprehensive understanding of sleep adaptations during altitude exposure. In addition, future investigations should consider controlling training load more carefully to distinguish the independent effects of hypoxic exposure and training stress on physiological and perceptual responses. Finally, studies including direct measures of erythropoietic adaptation, such as haemoglobin mass and reticulocyte responses, may help clarify the relationship between sleep, acclimatisation, and altitude-induced performance adaptations. Because the chronotype findings are the most novel part of this study, future research should focus on this area by combining objective sleep measures with athlete interviews, helping clarify the sleep issues seen in evening-type athletes and whether early morning wake-up times make them worse.

## 5. Conclusions

In this case series of world-class swimmers, sleep difficulty was descriptively higher during the first week at altitude, alongside changes in SpO_2_ and resting heart rate consistent with physiological acclimatisation. The observed association between chronotype and sleep difficulty appeared to be present before altitude exposure rather than emerging specifically during the camp. Given the small sample size, these findings should be regarded as preliminary. These findings generate testable hypotheses, particularly regarding chronotype as a screening tool, for future, adequately powered studies in elite athletic populations.

## Figures and Tables

**Figure 1:**
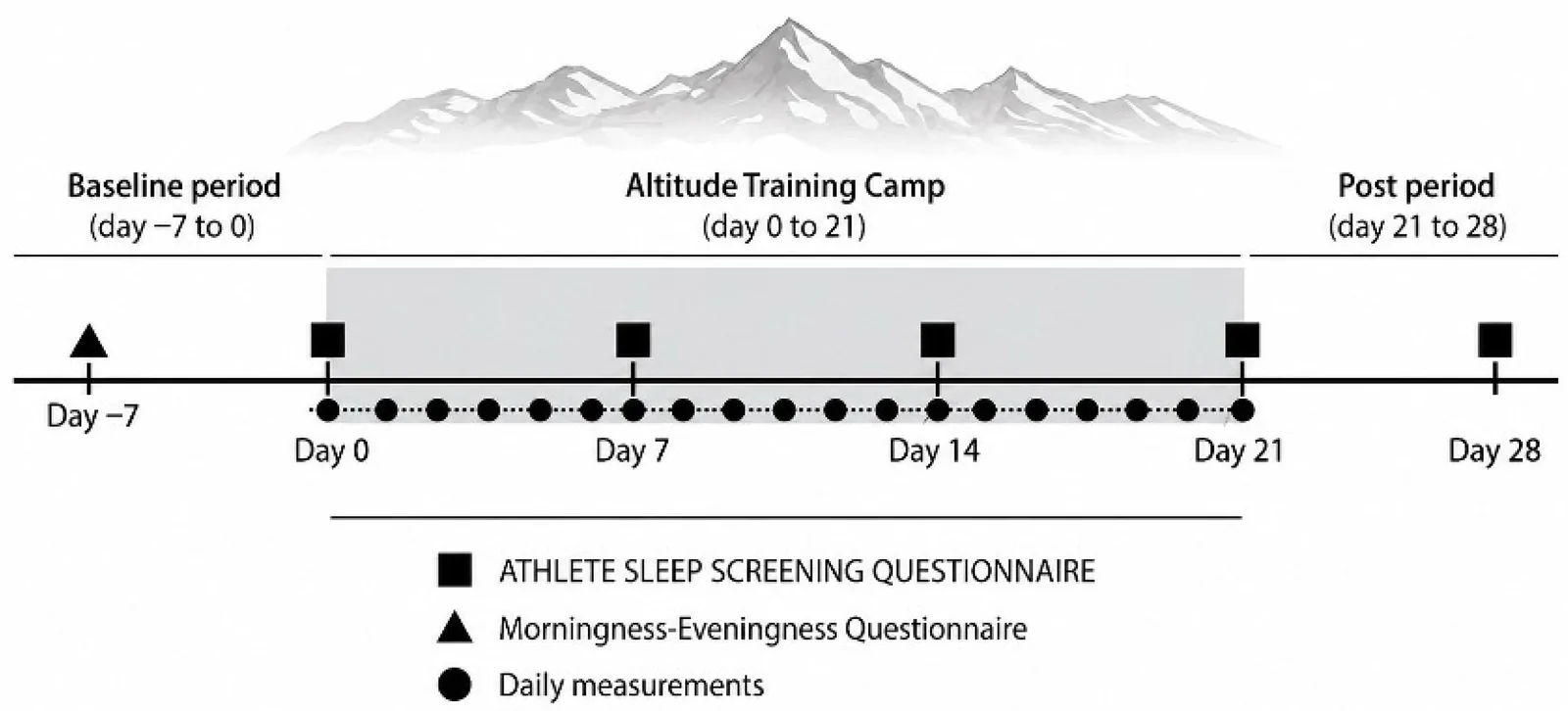
Study design and measurement schedule during the altitude training camp. Note: Study design and data collection timeline during the 3-week altitude training camp. ASSQ = Athlete Sleep Screening Questionnaire; MEQ = Morningness-Eveningness Questionnaire. Sleep difficulty was assessed using the ASSQ at baseline, at the end of each week during altitude exposure (weeks 1–3), and during the post-altitude phase. Daily monitoring during the altitude camp (days 0–21) included resting heart rate, SpO_2_, hydration status, perceived recovery, fatigue, and altitude-related symptoms. Training load was recorded following the final training session of each day.

**Figure 2:**
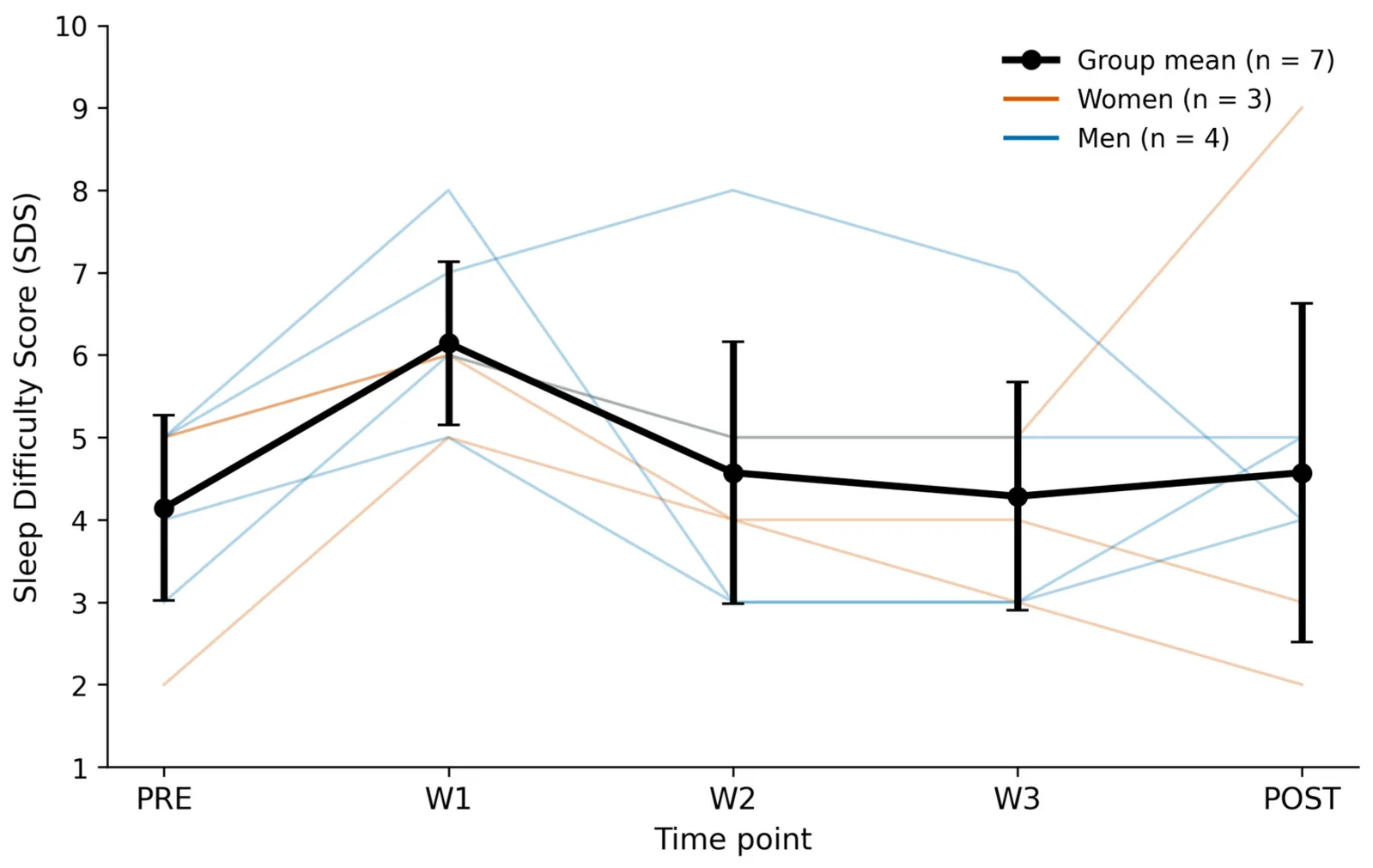
Evolution of Sleep Difficulty Score (SDS) before, during, and after the altitude training camp. Note: The thick black line represents the group mean (*n* = 7), with error bars indicating the 95% confidence intervals (95% CI). Thin lines represent individual athlete trajectories, coloured by sex (women in orange, men in blue). At PRE and week 1, two female athletes reported identical Sleep Difficulty Scores, resulting in overlapping lines. SDS = Sleep Difficulty Score, with higher scores indicating greater sleep difficulty; PRE = pre-altitude baseline; W1, W2, and W3 = weeks 1, 2, and 3 of the altitude camp, respectively; POST = post-altitude phase.

**Figure 3:**
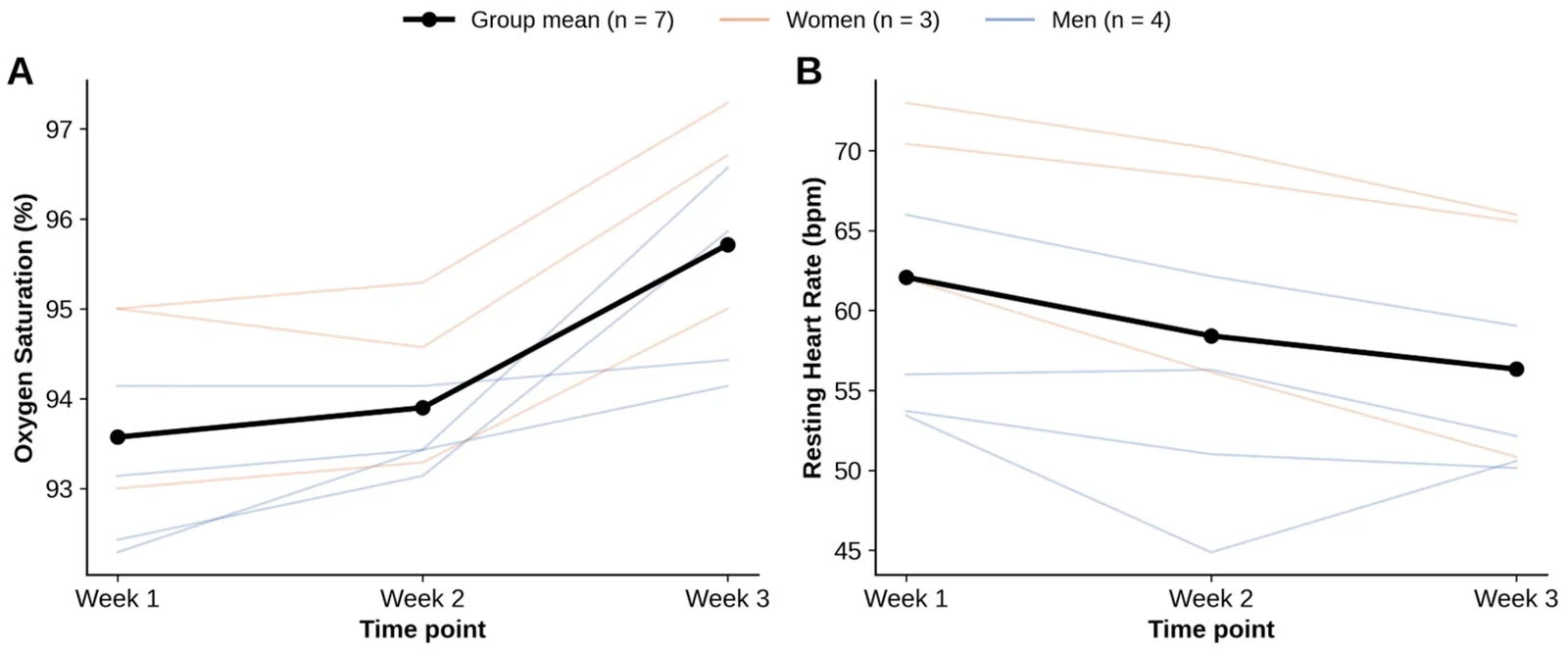
Evolution of physiological acclimatisation markers (SpO_2_ and resting heart rate) during the 3-week altitude training camp. Note. (**A**) Resting oxygen saturation (SpO_2_) and (**B**) resting heart rate (RHR) across the three weeks of altitude exposure. Thick black lines represent group means (*n* = 7), while thin grey lines indicate individual athlete trajectories. bpm = beats per minute.

**Table 1:** Spearman’s correlation coefficients between chronotype and sleep difficulty at different assessment points.

Assessment Point	*n*	Rho	*p*	95% CI
Pre-altitude	7	−0.87	0.014	[−0.98, −0.36]
Week 1 at altitude	7	−0.85	0.019	[−0.98, −0.27]
Week 2 at altitude	7	0.04	0.963	[−0.73, 0.77]
Week 3 at altitude	7	−0.17	0.714	[−0.82, 0.66]
Post-altitude	7	−0.37	0.419	[−0.88, 0.53]

Note: Data are presented as Spearman’s rank correlation coefficient (rho), two-sided exact permutation *p*-value, 95% confidence interval (95% CI), and sample size (*n*). Confidence intervals were calculated using Fisher’s z transformation. The Bonferroni-adjusted significance threshold for the five correlations was α = 0.010.

**Table 2:** Weekly evolution of peripheral oxygen saturation and resting heart rate during altitude exposure.

Variable	Week 1	Week 2	Week 3	χ^2^(2)	*p*
SpO_2_ (%)	93.57 ± 1.14	93.90 ± 0.80	95.71 ± 1.22	12.07	0.002
RHR (bpm)	62.08 ± 8.02	58.41 ± 9.10	56.33 ± 7.14	10.28	0.006

Note. Data are presented as mean ± standard deviation. SpO_2_ = peripheral oxygen saturation; RHR = resting heart rate; bpm = beats per minute. Overall differences across weeks were analysed using the Friedman test. Following a significant overall effect, pairwise comparisons were performed using two-tailed Wilcoxon signed-rank tests, with Bonferroni-adjusted *p*-values for the three pairwise comparisons. Pairwise test statistics, adjusted *p*-values, and effect sizes are reported in the text.

**Table 3:** Weekly evolution of athlete-reported metrics (altitude symptoms, fatigue, hydration, and perceived recovery) during the 3-week altitude training camp.

Variable	Week 1	Week 2	Week 3	x^2^(2)	*p*
Lake Louise Score	1.47 ± 0.51	1.96 ± 0.74	1.76 ± 0.53	4.69	0.09
TSF total	30.83 ± 1.44	28.52 ± 3.33	29.85 ± 3.15	5.43	0.06
Hydration	3.25 ± 0.66	3.10 ± 0.46	3.06 ± 0.39	0.58	0.75
Recovery	4.01 ± 0.71	4.30 ± 1.17	4.30 ± 0.88	0.28	0.87

Note: Data are presented as mean ± standard deviation. LLS = Lake Louise Score; TSF = Total Score of Fatigue. Hydration status was assessed using a urine color chart, and recovery was evaluated using a 0–10 rating scale. Differences across weeks were analyzed using the Friedman test.

**Table 4:** Weekly training load during the altitude camp.

Variable	Week 1	Week 2	Week 3
Volume (km)	57.93 ± 6.95	59.71 ± 5.35	38.21 ± 5.35
RPE	7.11 ± 0.41	5.67 ± 0.36	5.29 ± 0.28
sRPE-min	1441.12 ± 77.85	878.68 ± 57.76	901.69 ± 53.52

Note: Values are mean ± SD (*n* = 7). Volume corresponds to total weekly in-water training distance. RPE = rating of perceived exertion. sRPE-min represents the individual weekly mean of session training load; session-level sRPE-min was calculated as RPE × session duration (min) and expressed in arbitrary units (AU). Group sRPE-min values represent the mean ± SD of the individual weekly means.

**Table 5:** Pre- to post-camp changes in haematological and iron-status blood markers.

Variable	*n*	Pre-Altitude	Post-Altitude	*p*
Haemoglobin (g/dL)	7	14.37 ± 1.24	14.58 ± 1.09	0.438
Haematocrit (%)	7	43.38 ± 3.01	44.33 ± 2.73	0.313
Erythrocyte count (×10^6^/µL)	7	4.87 ± 0.39	4.88 ± 0.35	0.875
Serum iron (µg/dL)	7	124.50 ± 34.04	100.67 ± 38.80	0.313
Transferrin (mg/dL)	7	260.17 ± 36.73	261.67 ± 38.47	1
Transferrin saturation (%)	7	38.31 ± 10.53	31.90 ± 12.66	0.438
Ferritin (ng/mL)	7	121.20 ± 63.12	106.20 ± 55.77	0.438

Note: Data are presented as mean ± standard deviation. Pre- to post-altitude comparisons were analyzed using the Wilcoxon signed-rank test for paired samples (*n* = 7).

## Data Availability

The data presented in this study are available on request from the corresponding author. The data are not publicly available due to privacy and ethical restrictions.

## Supplementary Materials

The following supporting information can be downloaded at https://www.mdpi.com/article/10.3390/sports14100449/s1: Supplementary Table S1. Evaluation of the Sleep Difficulty Score (SDS) by sex; Supplementary Table S2. Chronotype (MEQ) by sex and distribution of chronotype categories by sex; Supplementary Table S3. Resting oxygen saturation and resting heart rate by sex; Supplementary Table S4. Lake Louise Score, TSF-10 total, hydration status, and perceived recovery by sex; Supplementary Table S5. Weekly training load by sex; Supplementary Table S6. Pre-to-post-altitude haematological and iron-status blood markers by sex; Supplementary Figure S1. Scatter plot showing the correlation between chronotype score (MEQ) and sleep difficulty (SDS) during the first week at altitude; Supplementary Figure S2. Heatmap of Spearman correlation coefficients between training load metrics and response variables across the 21-day altitude training camp.
