# Supplementary material for: Sleep, Chronotype and Physiological Acclimatisation During a 21-Day Altitude Training Camp in World-Class Swimmers
Source: Sports (Basel). 2026 Oct 11;14(10):449. doi: 10.3390/sports14100449 (PMC13709748; doi:10.3390/sports14100449)
Supplement: Supplementary file 1 [file sports-14-00449-s001.zip › sports-4555094-supplementary.pdf]

Table S1

Evolution of the Sleep Difficulty Score (SDS) by sex

Variable	Women	Men
Pre-altitude	4.00 ± 1.73	4.25 ± 0.96
Week 1	5.67 ± 0.58	6.50 ± 1.29
Week 2	4.33 ± 0.58	4.75 ± 2.36
Week 3	4.00 ± 1.00	4.50 ± 1.91
Post-altitude	4.67 ± 3.79	4.50 ± 0.58
Individual maximum value	6.67 ± 2.08	6.75 ± 1.50

Note: SDS = Sleep Difficulty Score, derived from the Athlete Sleep Screening Questionnaire (ASSQ); higher values indicate greater sleep difficulty. Values are mean ± SD (women, n = 3; men, n = 4).

Table S2

Supplementary Table S2. Chronotype (MEQ) by sex and distribution of chronotype categories by sex.

Variable	Women	Men
MEQ score	54.67 ± 6.66	51.50 ± 8.89

Distribution of chronotype categories by sex

Chronotype category (MEQ)	Women (n)	Men (n)
Extreme evening type	0	0
Moderate evening type	0	1
Intermediate type	2	2
Moderate morning type	1	1
Extreme morning type	0	0

Note: MEQ = Morningness-Eveningness Questionnaire. Higher scores indicate a stronger morningness tendency. No female participant was classified as extreme evening type or extreme morning type.

Table S3

Resting oxygen saturation and resting heart rate by sex

Variable	Women	Men
SpO2 Week 1 (%)	94.33 ± 1.15	93.00 ± 0.85
SpO2 Week 2 (%)	94.38 ± 1.01	93.54 ± 0.43
SpO2 Week 3 (%)	96.33 ± 1.19	95.25 ± 1.16
RHR Week 1 (bpm)	68.48 ± 5.75	57.28 ± 5.92

RHR Week 2 (bpm)	64.86 ± 7.61	53.57 ± 7.38
RHR Week 3 (bpm)	60.80 ± 8.64	52.97 ± 4.14

Note: SpO_2 = peripheral oxygen saturation; RHR = resting heart rate; bpm = beats per minute. Weekly values correspond to the average of daily measurements recorded within each week at altitude.

Table S4

Lake Louise Score, TSF-10 total, hydration status, and perceived recovery by sex

Variable	Women	Men
Lake Louise Score Week 1	1.33 ± 0.43	1.57 ± 0.61
Lake Louise Score Week 2	1.62 ± 0.66	2.21 ± 0.78
Lake Louise Score Week 3	1.52 ± 0.44	1.93 ± 0.59
TSF-10 total Week 1	30.62 ± 1.28	30.99 ± 1.73
TSF-10 total Week 2	27.39 ± 0.84	29.36 ± 4.41
TSF-10 total Week 3	29.90 ± 3.04	29.82 ± 3.70
Hydration Week 1	3.67 ± 0.58	2.93 ± 0.58
Hydration Week 2	3.34 ± 0.50	2.93 ± 0.41
Hydration Week 3	3.29 ± 0.29	2.89 ± 0.39
Recovery Week 1	3.57 ± 0.43	4.35 ± 0.74
Recovery Week 2	3.38 ± 1.30	5.00 ± 0.35
Recovery Week 3	3.86 ± 0.43	4.86 ± 0.93

Note: LLS = Lake Louise Score (revised) for acute mountain sickness symptoms; TSF-10 = Total Score of Fatigue; hydration status assessed via urine colour chart; perceived recovery assessed on a 0-10 scale based on the Total Quality Recovery Scale.

Table S5

Weekly training load by sex

Variable	Women	Men
Volume Week 1 (km)	59.17 ± 7.51	57.00 ± 7.51
Volume Week 2 (km)	60.67 ± 5.77	59.00 ± 5.77
Volume Week 3 (km)	39.17 ± 5.77	37.50 ± 5.77
RPE Week 1	7.00 ± 0.63	7.20 ± 0.24
RPE Week 2	5.76 ± 0.30	5.60 ± 0.43
RPE Week 3	5.20 ± 0.20	5.35 ± 0.34
sRPE-min Week 1	1429.25 ± 61.58	1450.02 ± 96.68

sRPE-min Week 2	896.99 ± 47.98	864.94 ± 67.45
sRPE-min Week 3	884.00 ± 38.12	914.95 ± 64.90

Note: RPE = rating of perceived exertion; sRPE-min = RPE × session duration in minutes (internal load). Volume corresponds to total weekly in-water training distance.

Table S6

Pre- to post-altitude haematological and iron-status blood markers by sex

	Women		Men	
**Variable**	**Pre-altitude**	**Post-altitude**	**Pre-altitude**	**Post-altitude**
Haemoglobin (g/dL)	13.73 ± 1.16	13.73 ± 0.75	15.00 ± 1.13	15.70 ± 0.65
Haematocrit (%)	42.00 ± 3.14	42.17 ± 1.75	44.77 ± 2.66	46.75 ± 1.10
Erythrocyte count (×10^6/µL)	4.61 ± 0.30	4.58 ± 0.19	5.13 ± 0.31	5.13 ± 0.15
Serum iron (µg/dL)	106.00 ± 24.04	81.00 ± 43.84	133.75 ± 37.36	110.50 ± 38.49
Transferrin (mg/dL)	295.50 ± 50.20	291.50 ± 53.03	242.50 ± 12.66	246.75 ± 25.29
Transferrin saturation (%)	28.00 ± 1.41	23.50 ± 16.26	45.18 ± 6.61	33.62 ± 10.36
Ferritin (ng/mL)	62.00 ± 29.70	48.50 ± 13.44	160.67 ± 41.06	143.25 ± 19.89

Note: Values are mean ± SD.

Supplementary Figure S1.

Scatter plot showing the correlation between chronotype score (MEQ) and sleep difficulty (SDS) during the first week at altitude.

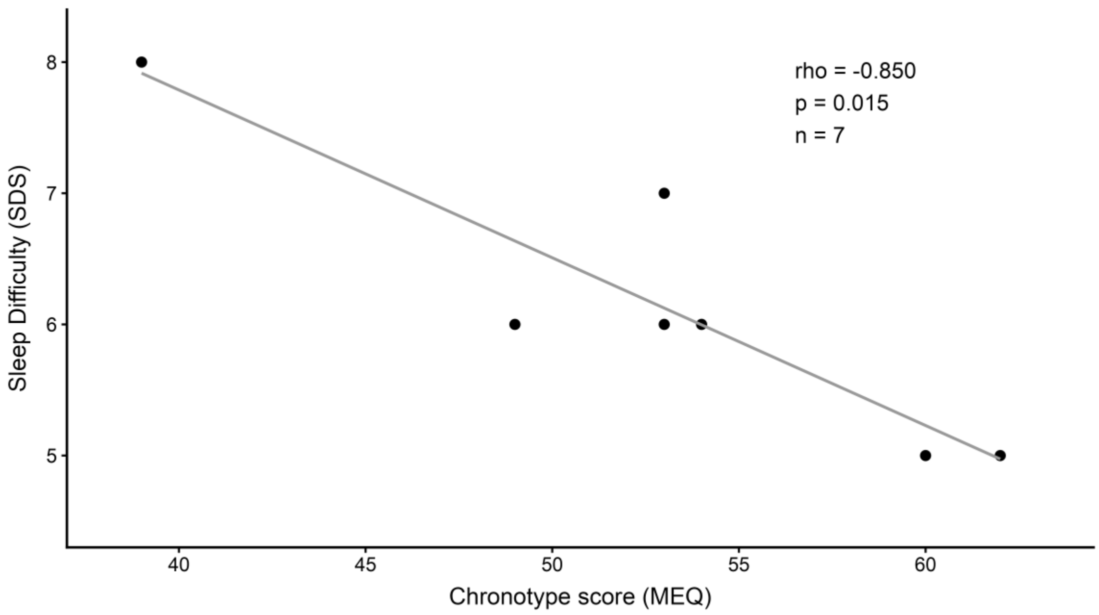

Note: The solid line represents the linear regression line. MEQ = Morningness–Eveningness Questionnaire, with higher scores indicating a stronger morningness tendency; SDS = Sleep Difficulty Score, with higher scores indicating greater sleep difficulty; rho = Spearman's rank correlation coefficient; p = statistical significance.

Supplementary Figure S2.

Heatmap of Spearman Correlation Coefficients Between Training Load and Response Variables Across the 21-Day Altitude Camp

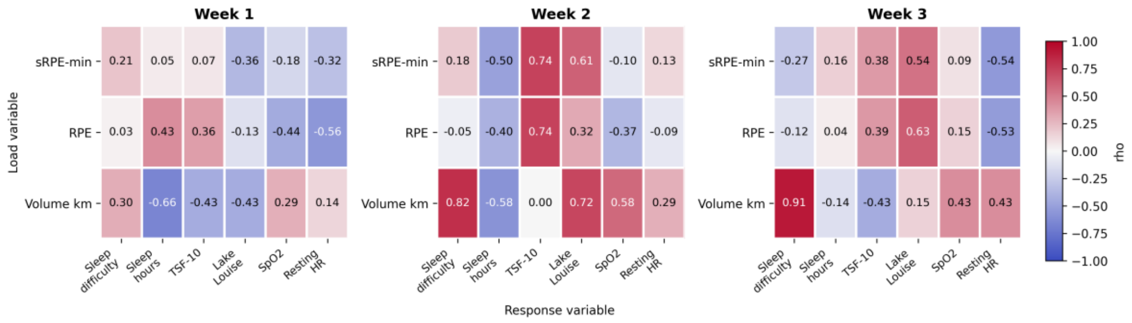

Note: The correlation matrices display Spearman's rank correlation coefficients (rho) separately for Week 1, Week 2, and Week 3. Warmer colors (red) indicate positive correlations, whereas cooler colors (blue) indicate negative correlations. sRPE-min = session rating of perceived exertion expressed in minutes; RPE = rating of perceived exertion; TSF-10 = Total Score of Fatigue (10-point scale). Given the large number of correlations examined relative to the sample size (n = 7), these results should be interpreted as exploratory and hypothesis-generating, rather than as evidence of meaningful physiological or perceptual relationships.
